# Digital wellbeing experience in the workplace: development and validation of the Work-Related Human Computer Interaction Questionnaire

**DOI:** 10.3389/fpubh.2026.1778040

**Published:** 2026-04-01

**Authors:** Desirée Estela Porcari, Davide Bottari, Iacopo Giribon, Gabriella Daneluzzi, Emiliano Ricciardi, Maria Donata Orfei

**Affiliations:** 1 Molecular Mind Laboratory (MoMiLab), IMT School for Advanced Studies Lucca, Lucca, Italy; 2 Neuroscience Lab, Intesa Sanpaolo Innovation Center S.p.A, Turin, Italy

**Keywords:** digital wellbeing, self-efficacy, attitude, technostress, human computer interaction, information and communication technology, validation

## Abstract

**Background:**

The rapid evolution of technology has triggered profound cultural, social, and psychological changes, along with a constant demand for human adaptation to new challenges. Digital wellbeing (DW) refers to the individual’s positive and healthy relationship with information and communication technology (ICT), characterized by feelings of comfort, support, safety, satisfaction, and low levels of stress during their interactions with ICT. However, DW can be threatened by ergonomic, organizational, and psychological issues, particularly in the workplace. Despite efforts to improve the objective conditions of user experience, the understanding of the role of psychological factors in HCI remains partially disregarded.

**Aims:**

This study aimed to validate a new multidimensional psychometric tool designed to assess the perceived quality of HCI in the workplace, the Work-Related Human Computer Interaction Questionnaire (wrHCI-Q).

**Methods:**

A sample of 1,198 employees of a large Italian banking group (52% females; age: 49.04 ± 8.7) underwent an online survey. Reliability, exploratory factor analysis (EFA), and confirmatory factor analysis (CFA) were performed.

**Results:**

The wrHCI-Q consisted of 35 items and a four-factor structure (technostress, self-efficacy, positive attitude, and HCI aversion), supported by the CFA indices.

**Conclusion:**

The wrHCI-Q is a new, valid, and reliable scale to catch some crucial human factors affecting the quality of HCI. It is expected to foster the understanding of the determinants of individual DWE and to shed light on the factors that undermine a healthy employee’s interaction with ICT.

## 1 Introduction

In the last decades, the rapid evolution of information and communication technologies (ICTs) has brought big changes in human activities, relationships, and environmental adjustments. As a result, individual and social well-being are heavily affected by the quality of interactions with ICTs (1).

The increasing role of ICT as part and parcel of human life and the related, relentless adjustment processes in human beings advocate a step forward in understanding HCI. In fact, the greater the evolution of ICT, the more challenging the interplay with it. On one side, most of the traditional approaches have focused on ergonomics (2–5), fatigue (6), and aging (7), as they represent traditional key aspects of workplace health and safety (8). This raising awareness has encouraged the development of different perspectives (9, 10). Recent trajectories of study extend research boundaries far beyond the mere user experience related to technologies, rather casting light on several factors playing a crucial role as determinants of DW, some of them rooted in ethics, values, social models, and even unaware expectations and concerns (11, 12). This wider perspective is fairly illustrated in the seven HCI grand challenges model (13), which provides a systematic and far-reaching glance at HCI, embracing topics such as creativity, ethics, and security. In terms of this model, the present study best deals with the challenge of human-environment interactions, underpinned by the awareness that technology and human beings are shaping a new environment with great opportunities, but also with increasing demands.

### 1.1 The paradox of HCI in the working context

The study of human-computer interaction (HCI) is crucial to understanding the profound changes in the working world. Undeniable advantages due to the enhancement of hybrid and remote work models and the benefits of the HCI on employees’ working conditions and satisfaction are highlighted in several studies (14, 15).

ICT has fostered a more flexible and efficient organization of work, facilitating communication and thus team activities and networking, shortening the execution of high-quality tasks, and encouraging decision-making processes (16). Despite the indisputable advantages of ICT in everyday life and in productive activities, growing evidence has cast light on the so-called “dark side” of HCI for both people and organizations: paradoxically, the advantages brought by ICT can turn into detrimental effects, jeopardizing workers’ quality of life (17–20). The increasingly massive use of ICT is accompanied by evidence of adverse effects: the boundaries between personal and professional life have become blurred, levels of stress, concentration, and job satisfaction are threatened by a lower sense of control and sense of techno-invasion (21). Moreover, the increased adoption of digital workplaces by companies to support work, communication, collaboration, and innovative practices inevitably tends to shape the way people feel and behave (22, 23). In light of this evidence, digital well-being (DW) is becoming a core issue of a new approach to work activities. DW refers to an individual’s ability to maintain a healthy, balanced, and sustainable relationship with digital technologies (24). The increasing complexity brought by technology and the related cultural revolution, including ethical and psychological issues, compels organizations to go beyond mere ergonomic guidelines. DW should be addressed as a function of the quality of digital interactions, perceived control over technology use, and the individual’s capacity for self-regulation (12), in other words, of the HCI.

Given these premises, DW in the workplace has to be conceptualized as a multidimensional and dynamic concept; for this reason, several theoretical models have been developed to draw an exhaustive framework. On one side, the minimization of techno-stressors such as techno-overload, techno-invasion, techno-complexity, techno-uncertainty, and techno-insecurity is considered essential to facilitate DW (25). On the other side, the awareness that DW is not granted merely by avoiding the harmful effects of ICTs has led to promoting a healthy adjustment to the HCI, including psychological growth, engagement, self-efficacy, and healthy social interaction (26).

Both of these approaches share the focus on the HCI, that is, the bidirectional relationship between the human being, and specifically the central nervous system and psychological organization, and the ICT. This is evident in the adaptation of the job demands-resources model (27, 28) to digital work environments, proposing that ICTs function both as job demands (e.g., constant connectivity, information overload, and technostress) and as job resources (e.g., enhancing productivity, autonomy, efficiency, and flexibility). Within this model, DW can be threatened by an imbalance in the HCI, that is, between demands and resources (29).

### 1.2 Assessment of HCI

Health surveillance procedures in the workplace refer to the systematic, ongoing process of monitoring workers’ health to detect early signs of work-related diseases, evaluate exposure-related health risks, and implement preventive or corrective actions (30, 31). Integrating DW into the organizational safety culture contributes to protecting employees’ psychophysical integrity. Despite this evidence, to the best of our knowledge, in the literature, the existing questionnaires do not satisfy these requirements fully. Very few scales were specifically designed for the working context, and the existing measures tend to mainly address technostress, taking into account traditional techno-stressors and conceptualizing the individual as a mere ICT user, thus failing to capture the higher complexity of the HCI evolution.

### 1.3 ICT and bank work activities

The digitalization in the banking context started in the sixties and made bank activities more and more technology-oriented, and there is evidence of higher levels of employee satisfaction and effectiveness due to the growing role of ICT, especially in developing countries (32). Despite this, the dark side of occupational stress has risen in the banking sector due to increased workload, technological advancements, performance pressure, and changing customer expectations (33). After the COVID-19 pandemic, in Italy, in the banking context, smart working has been incentivized, except for branch employees. On one side, many workers welcome this impulse to remote work, as it facilitates flexibility and lower costs. However, smart working poses additional HCI challenges. A higher number of interruptions during work activities, a greater overlap between private and work life, a decrease of vis a vis interaction with Ipsen et al. (34) may make bank employees less productive and more stressed and lead to a lower engagement in the level of team spirit (35–38). Further, such a massive engagement in HCI requires higher levels of job autonomy, with a greater demand for individuals to decide how, how often, and when they will use ICT and carry out their tasks. The role of job autonomy in HCI is still controversial in the literature (39).

Finally, in bank branches, the ongoing direct contact with clients may significantly jeopardize technostress, representing an additional element of pressure and demand, primarily due to workload, time pressure, performance targets, and technological changes (33).

### 1.4 Aims of the study

The present study aims to introduce and validate the Work-Related Human Computer Interaction Questionnaire (wrHCI-Q), a short and easy-to-use questionnaire, to assess HCI specifically in the Italian banking context.

Specifically, the wrHCI-Q was designed to serve two purposes. First, it was developed to provide a suitable answer according to the cultural shift taking place in the study of HCI, and thus moving from conventional interaction and user interfaces toward a human-centric challenging adjustment (40).

Second, the wrHCI-Q is supposed to overcome some of the above-mentioned gaps and limitations of previous assessment tools, providing high flexibility and manageability in routine health surveillance procedures, and good applicability to various ICT devices and tools.

The need to meet these requirements suggested moving toward three dimensions that were shown to play a relevant role in conditioning HCI in the workplace, namely, technostress, ICT attitude, and ICT self-efficacy. These dimensions have gained growing attention as, on one side, they are expected to determine if ICTs are experienced as resources or stressors in the workplace, while on the other side, they reflect psychological and cultural elements.

#### 1.4.1 Technostress

Technostress (TS) is defined as any negative impact on attitudes, thoughts, behaviors, or body physiology that is caused either directly or indirectly by technology (41). Information overload, constant interruptions, and complex digital interfaces increase mental effort and deplete attentional resources, contributing to cognitive overload, digital fatigue, work–family conflict, emotional exhaustion, poor sleep quality, and reduced performance (42–46). Several studies describe the detrimental impact of technostress on workers, and point out the relationship with phenomena such as burnout, depression, anxiety, and perceiving social pressure to be constantly available or connected and to prove capabilities at internet multitasking (47). Salanova et al. (41) stress the concept that ICTs are mere instruments; thus, they are not harmful per se, but rather the quality of HCI determines a backlash occurring when job demands do not match a worker’s capabilities, resources, and needs (48).

Most of the questionnaires concerning technostress reflect the classical models of the construct (25, 45). Thus, despite their value and validity, they catch a limited range of facets, neglecting the wider phenomenon of HCI. Other questionnaires emphasize the risk factors of digital stress, such as time pressure, uncertainty, response expectations, 24/7 availability, ineffective communication, and workload (49–52). Actually, they mainly focus on the stressor role of ICT, disregarding its potential positive contribution and failing to investigate which personal resources, such as sense of control, self-confidence, or openness to change, can foster a coping response against ICT-related distress, resulting into healthier psychological, behavioral and physiological outcomes for the individual (53, 54). Molino et al. (55) validated the Italian version of the Technostress Creators Scale (25, 45), anchored to the assumption that technostress occurs when environmental demands exceed individuals’ resources and manifests psychological and behavioral responses to stressors present in the work environment. Specifically, the tool stems from a more traditional approach to HCI, centered on three common techno-stressors, namely techno-overload, techno-invasion, and techno-complexity, but was not designed to pick the challenging interaction demands with ICT in the workplace.

More recent questionnaires have been developed to capture both positive and negative aspects of technology use (56, 57). However, despite the ease of use of these tools and their attempt to assess a wider range of dimensions, they are characterized by methodological limitations, primarily related to the characteristics of the samples, resulting in low generalizability of the results. Finally, a few questionnaires challenge specific psychological factors, such as ICT attitude (58) and ICT self-efficacy (59). They are worthwhile as they challenge issues that are not frequently addressed. However, they are mostly focused on Internet use, and the validation was run on college students; thus, not only do they provide limited information, but also, their generalizability is rather circumscribed.

#### 1.4.2 ICT attitude

According to the APA dictionary,^1^ attitude is a relatively enduring and general evaluation of an object, person, group, issue, or concept on a dimension ranging from negative to positive. Attitudes provide summary evaluations of target objects and derive from specific beliefs, emotions, and past behaviors associated with those objects (60). Positive and negative attitudes can have a significant influence on behavior in various situations, as they include opinions, emotions, perceptions, beliefs, expectations, values, and intentions. Each attitude is defined by three components that is, affective, behavioral, and cognitive (61). Although enduring, attitudes are susceptible to change when the intervention strategy is focused on moderating these three dimensions (62). In this theoretical perspective, ICT attitude denotes an individual’s general disposition toward the use of ICTs, encompassing cognitive beliefs, emotional responses, and behavioral intentions related to digital tools and environments (63, 64). A positive ICT attitude is typically associated with greater confidence and reduced anxiety (65–67), and specifically a positive perception and attitude toward ICT is one of the main factors influencing ban employee effectiveness (68) while negative ICT attitudes turned out to be one of the main causes of resistance toward computer technology, thus jeopardizing user satisfaction, frequency of use, and students’ academic performance (69).

#### 1.4.3 ICT self-efficacy

Another psychological dimension frequently highlighted as strongly impacting HCI is self-efficacy. According to the social cognitive theory, self-efficacy can be defined as the generative capability in which cognitive, social, and behavioral subskills must be organized into integrated courses of action to serve innumerable purposes (70). The role of perceived self-efficacy is supposed to be more effective than actual skills, as it affects and orients choices of behavioral activities, effort expenditure, persistence, frustration tolerance, and task performance (71–73). Thus, when applied to the interaction with ICT, self-efficacy can be conceived as one’s perception not merely of his/her capabilities regarding specific computer-related knowledge and skills, but also of solving problems, facing unexpected hazards, and adapting to changes. In this acceptation, ICT self-efficacy embraces techno-uncertainty, a psychological reaction mediated by anxiety and sense of control (74). Considering that self-efficacy is related to stress and anxiety and even to burnout (75), the lower the sense of self-efficacy, the greater the sense of threat perceived when interacting with ICT. Previous studies highlighted that higher levels of ICT self-efficacy were positively related to greater productivity, satisfaction, and ability to cope with ICT (76). Interestingly, some authors shifted from the original concept of techno-complexity illustrated in the theory of techno-stressors and techno-creators (25, 45) to the construct of ICT self-efficacy, best dealing with a sense of lack of control on technology (77).

## 2 Materials and methods

### 2.1 Study design

A cross-sectional web-based study was performed. All participants were provided with a detailed description of the experimental procedures and required to give consent before participating in the study. The survey was anonymous since each participant was assigned an alphanumeric code. We collected data from 8th to 22nd September 2023, and the survey was evenly distributed across the national territory. The study was conducted following the ethical standards laid down in the 1964 Declaration of Helsinki and under a protocol approved by the Joint Ethical Committee for Research of Scuola Normale Superiore, Scuola Superiore Sant’Anna, and IMT School for Advanced Studies Lucca (Protocol No. 34/2022).

### 2.2 Participants

A panel of 5,676 employees of a large Italian banking group whose daily work activities imply ICT use was invited to participate in an online survey. Inclusion criteria were: (a) age higher than or equal to 18 years old, (b) Italian mother tongue or high-level knowledge of the Italian language, and (c) use of ICTs (personal computer, tablet, smartphone, software, and messaging and video calling tools) during one’s everyday work activity. The subjects were part of different business units (BU), i.e., central structures, branch, new concept branch, and digital branch. They covered several roles in the bank, i.e., responsible, not responsible, network coordinator, and operational coordinator of activities. Moreover, the participants were allocated to the whole national territory. From the initial panel, 1,198 participants (52% females; age: 49.04 ± 8.7) filled in the survey.

### 2.3 Measure development

The aim was to develop an original questionnaire to validate in the Italian working context, investigating three dimensions: (a) technostress, (b) ICT self-efficacy, and (c) ICT attitude. For the very first draft of the questionnaire, we referred to three existing questionnaires, selecting the suitable items, translating into Italian where necessary, and rephrasing the content to make them suitable for the working contexts. For the first dimension, technostress, we included the questions from the 17-item Work-Related Technostress Questionnaire (WRT-Q) (78). The WRT-Q was recently developed to cover the limitations of previous scales, such as excessive length, lack of focus on behavioral manifestations of TS, and poor suitability for routine occupational surveillance across different roles and ICT tools. The WRT-Q aimed to increase the understanding of workers’ TS and is rooted in work-related stress research and in the latest theoretical models of technostress. It showed good validity and reliability indices and encompasses four factors: quality of work life, cognitive overload, intrusion, and psychophysical stress.

To investigate the ICT attitude, we referred to the 18-item Internet Attitude Scale (58), a multidimensional instrument designed to measure high school students’ attitudes, feelings, and evaluative judgment about using the Internet in educational and everyday contexts. We performed a back-translation from English to Italian and partially rephrased the items to adapt them to the aims of the study, in particular to address an adult working population and to consider ICT beyond mere Internet use.

Finally, ICT self-efficacy was investigated by translating into Italian and adapting to the ICT and workplace context the 8-item Internet Self-Efficacy Scale (59). The original questionnaire was made of individuals’ judgments of their ability to use the Internet to produce overall attainments and was underpinned by social cognitive theory.

All the above-mentioned scales were selected among others for their good characteristics of validity and reliability, and for their potential adaptability to other contexts and developments.

Both original questionnaires—the 18-item Internet Attitude Scale and the 8-item Internet Self-Efficacy Scale—underwent a forward-backward translation process. First, they were translated into Italian by a native English speaker with advanced proficiency in Italian and experience in the field. Subsequently, a second independent translator, who was blind to the original version, translated the Italian version back to English. The original and back-translated versions were then compared to identify and resolve any discrepancies, ensuring that the Italian version accurately reflected the meaning and intent of the originals.

The first draft of the questionnaire consisted of 43 items. Three experts in neuroscience, psychology, and behavioral economics oriented to working contexts (MO, DB, and ER) assessed the content validity of each item on a 4-point scale as follows: 1 (irrelevant), 2 (equivocal or redundant), 3 (relevant but needs minor revisions), and 4 (relevant and clear). The threshold for each item was set at equal to or higher than 3 for all three judges. As a result, 4 items were deleted. The remaining items showed an inter-judge reliability of 0.8.

The final questionnaire consisted of 39 self-administered items; each rated on a 4-point Likert scale (0 = not at all/never/fully agree to 3 = completely/always/fully disagree). The global score was obtained by adding each item and ranged from 0 to 117, where higher scores indicated greater difficulties in HCI in the workplace. Five reverse items were included.

### 2.4 Statistical analyses

Data were analyzed using the open-source statistical software JASP. The Cronbach’s alpha and McDonald’s omega tests (>0.80) were performed with inter-item correlation to test questionnaire reliability; items with an over-threshold correlation can result in redundancy and multicollinearity, therefore, items with high correlation (>0.70) were removed. Keiser–Meyer–Olkin (KMO; >0.60) and Bartlett’s sphericity tests (*p* < 0.05) were used to evaluate the adequacy and suitability of the sample before performing the factor analysis. Exploratory factor analysis (EFA) employed principal components analysis with oblique rotation (oblimin) was performed and enforced a four-factor solution to test the theoretical structure of wrHCI-Q. We adopted the oblimin or oblique rotation because it is more appropriate when the questionnaire items are not supposed to be orthogonal, that is, independent of each other, as in this case. To ascertain the factor solution, CFA was performed. The goodness-of-fit of the model was based on: S-B *χ*^2^/df *p* > 0.05, CFI >0.90, IFI >0.90, GFI >0.90, SRMR <0.08, and RMSEA between 0.05 and 0.08. The sample was randomly distributed in two subsamples (Group 1, *N* = 625, and Group 2, *N* = 573) to perform EFA and CFA, respectively.

## 3 Results

Table 1 shows the demographic characteristics of the two subsamples. The chi-square test highlighted no significant differences in gender distribution, and the independent sample *t*-test was also not statistically significant for age.

**Table 1:** Socio-demographic characteristics of subsamples.

Variable	Group 1 *N* = 625	Group 2 *N* = 573		
	*N* (%)	*N* (%)	*χ*^2^ (or *t*)	*p*
Gender			3.630	0.057
Women	342 (54.7)	282 (49.2)		
Men	283 (45.3)	291 (50.8)		
Age	49.03 ± 9.0	49.05 ± 8.4	0.043	0.966

### 3.1 Reliability

The questionnaire was found to be highly reliable (Table 2). The internal consistency reliability showed items with a correlation above the acceptable threshold. The higher correlation between items 1 and 2 (*r* = 0.861) and items 1 and 3 (*r* = 0.804) suggested they were redundant in content. Thus, item 1 was removed. Specifically, we kept items 2 and 3 as they inquire about the ability to understand terms and functionality of the hardware and software of ICTs. The questionnaire showed high reliability even if item one was removed (Table 2).

**Table 2:** Reliability of the wrHCI-Q.

wr-HCI	Cronbach’s alpha	McDonald’s omega
39-items version	0.884	0.894
38-items version	0.936	0.937
35-items version	0.936	0.937

### 3.2 Exploratory factor analysis

In the first sample (*N* = 625), the KMO value (0.940) and Bartlett’s sphericity test (*χ*^2^ = 13233.680 *p* < 0.001) showed that the data were suitable for factor analysis. To explore the factorial structure of the wrHCI-Q, all 38 items of the instrument underwent exploratory factor analysis (EFA) with oblique rotation (oblimin), which allows correlation between the latent factors. Out of 38 items, three items (i.e., 17, 36, and 38) did not statistically match and did not reach the acceptable factor loading index (≥|0.40|) (79). Thus, the final version of the questionnaire was composed of 35 items and four factors, with a global score ranging from 0 to 105 and 65% of the variance explained by these four factors (Table 3).

**Table 3:** EFA with oblique rotation (oblimin) (*N* = 625).

Item	Factors			
	Technostress	Self-efficacy	Positive attitude	HCI aversion
Item 23	**0.654**	−0.101	0.048	−0.003
Item 24	**0.607**	−0.120	0.117	0.132
Item 25	**0.689**	−0.085	0.101	0.048
Item 26	**0.789**	0.014	−0.004	−0.049
Item 27	**0.679**	0.015	−0.027	0.015
Item 28	**0.656**	−0.034	−0.065	−0.199
Item 29	**0.656**	0.114	0.023	−0.007
Item 30	**0.435**	0.282	−0.086	0.256
Item 31	**0.546**	0.171	−0.032	−0.129
Item 32	**0.569**	0.098	−0.001	−0.090
Item 33	**0.683**	0.061	0.037	0.084
Item 34	**0.461**	−0.019	0.100	0.373
Item 35	**0.612**	−0.030	0.024	0.103
Item 37	**0.687**	−0.031	0.054	0.001
Item 39	**0.731**	0.060	−0.058	0.058
Item 2	0.073	**0.797**	−0.011	−0.014
Item 3	0.044	**0.818**	−0.057	−0.046
Item 4	−0.013	**0.852**	0.028	0.002
Item 5	0.001	**0.871**	−0.035	−0.047
Item 6	0.032	**0.739**	0.063	0.032
Item 7	0.038	**0.673**	0.104	0.087
Item 8	−0.056	**0.637**	0.109	0.125
Item 13	−0.074	**0.581**	0.177	0.144
Item 16	−0.035	**0.531**	0.067	0.045
Item 19	−0.029	**0.481**	0.174	0.229
Item 10	0.104	0.170	**0.629**	−0.122
Item 15	0.021	0.176	**0.704**	−0.026
Item 18	0.019	−0.027	**0.783**	−0.011
Item 21	0.011	0.033	**0.694**	0.104
Item 22	−0.021	−0.060	**0.766**	0.008
Item 9	0.041	0.002	0.086	**0.608**
Item 11	−0.022	0.276	−0.104	**0.677**
Item 12	0.074	0.069	−0.041	**0.641**
Item 14	0.040	−0.063	0.112	**0.694**
Item 20	0.082	−0.045	0.016	**0.486**
Item 17	0.213	−0.055	0.261	0.272
Item 36	0.338	0.204	−0.016	0.211
Item 38	0.316	−0.004	−0.259	0.024

The items that reach the acceptable factor loading index (≥|0.40|) are highlighted in bold.

**Figure 1:**
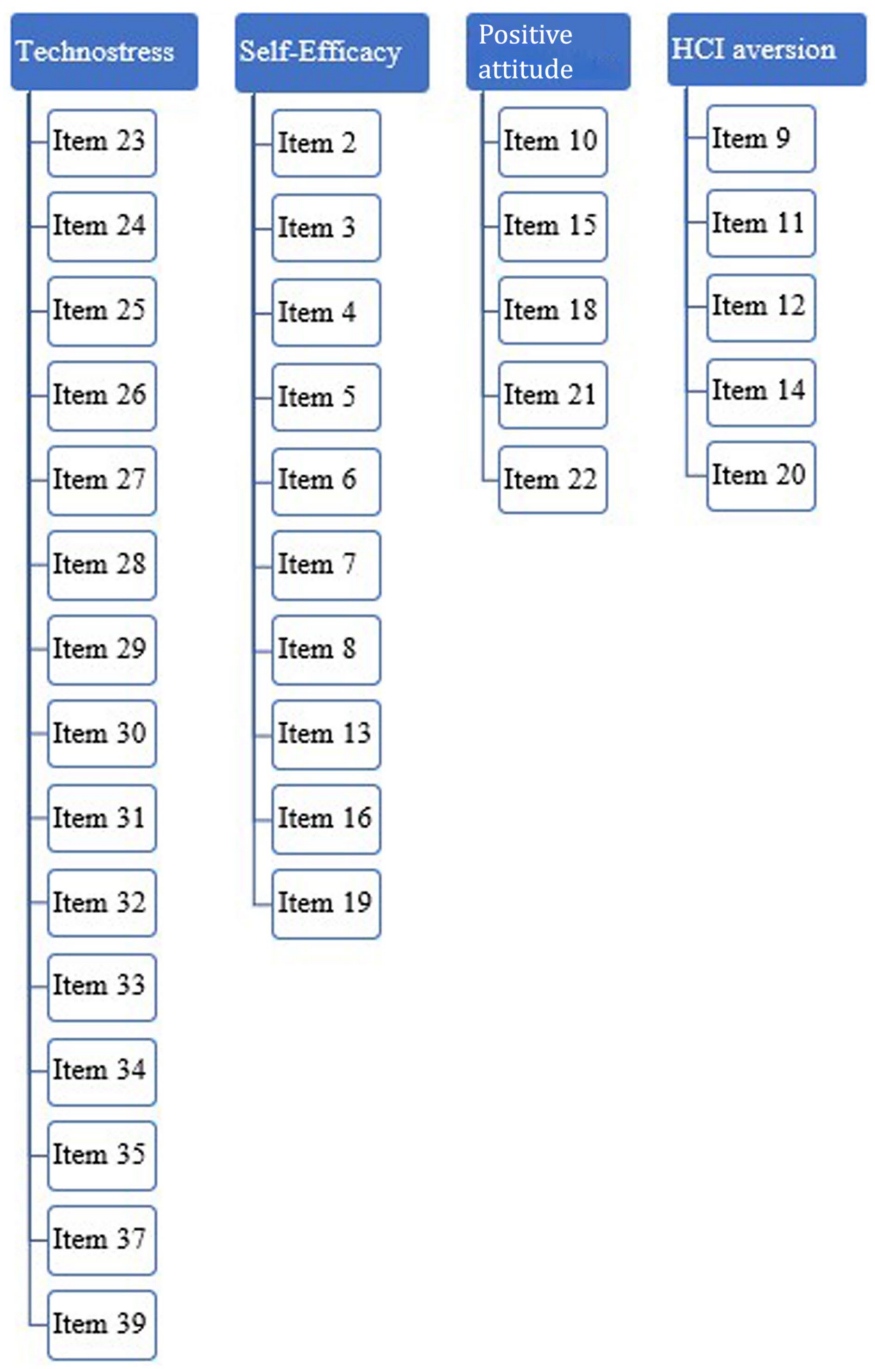
The path diagram of the four-factor model.

The factors resulted positively correlated; the factor correlations matrix of EFA is shown in Table 4 based on the items’ contents, factors were renamed respectively: technostress, self-efficacy, positive attitudes, and HCI aversion (Figure 1). The Italian version of the wrHCI-Q is reported in Appendix A, while a not validated English translation is reported in Supplementary material.

**Table 4:** Correlation matrix of the factors in the EFA model (*N* = 625).

Factor	Technostress	Self-efficacy	Positive attitude	HCI aversion
F1	1			
F2	0.311	1		
F3	0.290	0.376	1	
F4	0.383	0.442	0.333	1

F1 = Technostress; F2 = Self-efficacy; F3 = Positive attitude; F4 = HCI aversion.

### 3.3 Confirmatory factor analysis

In the second subsample (*N* = 573) the four-factor solution of wrHCI-Q was re-examined using CFA to determine its model fit. Before conducting CFA, skewness and kurtosis were performed; the distribution was approximately normal (skewness = 0.418; kurtosis = 0.206). The chi-square goodness of fit was statistically significant (*χ*^2^ = 1307.016 df = 554, *p* < 0.001). For the model of the wrHCI-Q, other multiple indices were used to judge the overall goodness of fit: CFI = 0.974; IFI = 0.974; GFI = 0.968; RMSEA = 0.049; and SRMR = 0.067. All the indices were within acceptable ranges, which means that the four factors obtained from EFA were validated and the wrHCI-Q had a high goodness of fit. There was a positive correlation between the factors, with estimates ranging from *r* = 0.365 to *r* = 0.603, and there was a significant relationship among the factors (*p* < 0.001) (Table 5). Descriptive statistics (means and standard deviations) of wrHCI-Q total score and its dimensions in the overall sample are riported in Table 6.

**Table 5:** Correlation matrix of the factors in the CFA model (*N* = 573).

Factor	Technostress	Self-efficacy	Positive attitude	HCI aversion
F1	1			
F2	**0.365**	1		
F3	**0.434**	**0.545**	1	
F4	**0.525**	**0.603**	**0.434**	1

Significant values (*p* < 0.001) are highlighted in bold. F1 = Technostress; F2 = Self-efficacy; F3 = Positive attitude; F4 = HCI aversion.

**Table 6:** Socio-demographic characteristics of wrHCI-Q.

Variable	*N* = 1,198 (M ± SD)
wrHCI-Q total	35.72 ± 15.60
Technostress	13.19 ± 7.82
Self-efficacy	16.50 ± 7.43
Positive attitude	4.40 ± 3.07
HCI aversion	2.92 ± 2.77

## 4 Discussion

The main aim of this study was to develop and validate a new, multifaceted, and manageable Italian questionnaire to assess the quality of adjustment to HCI in the workplace, specifically in an Italian banking context. To achieve this goal, a literature review was conducted on the DW framework, determinants, and existing psychometric tools, and three key dimensions were initially selected (technostress, ICT self-efficacy, and ICT attitude). As a result, 43 items were generated to measure and describe the core point of each dimension identified on a 4-point Likert scale. The result of EFA and CFA provided a questionnaire consisting of 35 final items and four factors, namely technostress, self-efficacy, positive attitude, and HCI aversion, supporting the idea that DW is a multidimensional and dynamic concept (24, 80); moreover, the resulting version of the wrHCI-Q showed high internal consistency.

### 4.1 Practical implications

To the best of our knowledge, this is the first Italian questionnaire to assess work-related HCI from a multidimensional perspective, going beyond the mere concept of technostress, thus providing relevant information to individuals and organizations to optimize HCI. The originality of our tool lies in the multifaceted nature and thus in the complex nature the factors affecting human adjustment to the ICTs, in particular: (a) the adverse emotional effects of technostress regardless of the kind of job, role, and ICT adopted in everyday work life; (b) personal belief in self-efficacy using ICT at work, and (c) positive attitude and HCI aversion in the workplace. The wrHCI-Q aims at eliciting aware and possibly even unaware aspects intrinsic to the relationship between the human nervous central system and technology. Indeed, the existing questionnaires are underpinned by classical theories on technostress, mainly focusing on technostress creators and inhibitors (45). Most of these scales include items formulated to investigate the consequences of organizational mismanagement, ergonomics, or difficulties in regulating the use of technology, that is, issues best rising in a previous stage of technology evolution. Differently, the wrHCI-Q represents the attempt to focus not only on the mis-use of ICT, but also on the mis-interaction with ICT. A similar approach can be found in the work of Castillo et al. (77), whose French translation and validation of the Technostress Creators and Inhibitors Scale, although still fitting to the original model of technostress, led to some adjustments to the construct of the scale, best focused on self-efficacy and self-control. The wrHCI-Q can be considered a further step forward, focusing on the human reactions elicited by the demands of a more and more interactive technology.

Our work highlights the need to enhance self-awareness in the relationship with ICTs, which in turn is related to self-control. Self-control is generally defined as the skill to resist immediate impulses or habits, enabling individuals to inhibit undesirable behaviors and prioritize the pursuit of long-term goals (81, 82). In the context of HCI, managing and regulating one’s digital interactions may be critical, allowing individuals to align their digital experiences with personal values and goals (11, 83).

In addition to this, organizations should adopt explicit digital well-being policies, including training programs on technology use and self-regulation, initiatives supporting the right to disconnect, guidelines for asynchronous communication, and digital etiquette norms. Such measures can increase employees’ sense of control, ICT attitude, and enhance productivity. Digital literacy, which includes both objective and perceived competence in technological concepts, is crucial for managing ICTs. Subjective digital literacy has been shown to positively influence DW, as it plays an essential role in helping people to evaluate the accuracy and credibility of information (11).

Managers should be trained to recognize signs of distress and promote healthy digital habits. A workplace culture that respects personal and digital boundaries contributes to greater psychological safety and engagement (55).

Likewise, workplace technologies should be developed according to well-being supportive design principles (84), emphasizing simplicity, transparency, personalization, and user control. Promoting digital well-being is not only a health-related initiative but also a sustainability strategy: balanced and intentional technology use could contribute to reducing turnover, improving organizational environment, and supporting long-term productivity.

### 4.2 Limitations

However, some limitations of the research must be highlighted. First, the study focused exclusively on Italian bank employees, thus circumscribing a relatively specific application context. Despite these considerations, the heterogeneity of mansions, roles, and business units represented in our sample, as well as the variety of ICTs used in everyday working routine, contributes to providing a fairly comprehensive variety of situations and job activities. Future research to properly extend the validity of our questionnaire in different work contexts is burn-out required.

Second, in our study, we did not include tools or questionnaires assessing neighboring phenomena; thus, we could not perform analyses of the convergent and/or discriminant validity. These additional points were beyond the scope of the present study, though they may serve as valuable directions for future research aimed at further strengthening the validity of the wrHCI-Q. Future studies could deepen the generalizability of results by comparing samples drawn from different populations and, where possible, conducting invariance analysis as well as its convergent and/or discriminant validity, both with other pre-existing tools and with other potentially related measures, particularly those linked to occupational stress and attitudes toward ICT use. Third, our questionnaire does not specifically address very recent technological challenges as artificial intelligence. Although this issue can be embedded in the topics investigated by the wrHCI-Q, future studies could further develop the scale to include artificial intelligence peculiarities.

### 4.3 Future studies and conclusions

Workplace DW experience arises from the interplay of individual (digital self-regulation, digital literacy, coping strategies), organizational (policies supporting disconnection rights and well-being culture), and technological factors (usability, interface design, notification management). This study advances the assessment of work-related HCI by developing a new Italian valid instrument tailored to the banking context, and effective for health surveillance screening. Future research should aim to validate the tool in other occupational settings. Our study is expected to stimulate eventual additional studies on the issue and promote further reflections and actions to facilitate human well-being in the workplace.

In conclusion, we consider our work a step ahead in the understanding of the adjustment process to HCI and advocate further studies to cast light on the complex intersection between several dimensions that may contribute to a healthy HCI in the workplace, to enhance quality of life as related to one of the most remarkable technological revolutions of the modern era.

## Data Availability

The raw data supporting the conclusions of this article will be made available by the authors, without undue reservation.

## Footnotes

1
https://dictionary.apa.org
