# Supplementary material for: Digital wellbeing experience in the workplace: development and validation of the Work-Related Human Computer Interaction Questionnaire
Source: Front Public Health. 2026 Apr 1;14:1778040. doi: 10.3389/fpubh.2026.1778040 (PMC13081781; doi:10.3389/fpubh.2026.1778040)
Supplement: Supplementary file 1 [file Supplementary_file_1.docx]

The questionnaire consists of statements describing different opinions, behaviors, attitudes, and feelings toward Information and Communication Technology (ICT) in the workplace (both devices, such as computers, tablets, smartphones, and tools such as work dashboards, messaging and video calling tools, business software, management software, etc.).

Please indicate how much each of the following 6 statements reflects your feelings and opinions.

1. I feel confident in understanding terms and words related to ICT software

a) Not at all

b) A little

c) Somewhat

d) A lot

1. I feel confident in describing the functionality of ICT hardware

a) Not at all

b) A little

c) Somewhat

d) A lot

1. I feel confident in solving any problems when using ICT

a) Not at all

b) A little

c) Somewhat

d) A lot

1. I feel confident in my ability to understand the reason for a program malfunctioning on a personal computer

a) Not at all

b) A little

c) Somewhat

d) A lot

1. I feel confident using ICT to collect and/or process data

a) Not at all

b) A little

c) Somewhat

d) A lot

1. I feel confident in learning new skills, as well as new ICT functions and programs

a) Not at all

b) A little

c) Somewhat

d) A lot

Please indicate how much you agree or disagree with each of the following 14 statements

1. In most cases, I can learn the things I need to use ICT on my own

a) Strongly disagree

b) Somewhat agree

c) Agree

d) Strongly agree

1. I tend to avoid using ICT if I may appear clumsy and inexperienced

a) Strongly disagree

b) Somewhat agree

c) Agree

d) Strongly agree

1. ICT enables work to be carried out in a more interesting and creative way

a) Strongly disagree

b) Somewhat agree

c) Agree

d) Strongly agree

1. I need someone with experience to help me use ICT

a) Strongly disagree

b) Somewhat agree

c) Agree

d) Strongly agree

1. When I have to use ICT, I am afraid of causing irreversible damage

a) Strongly disagree

b) Somewhat agree

c) Agree

d) Strongly agree

1. If I have problems while using ICT, I am usually able to resolve them by myself in one way or another

a) Strongly disagree

b) Somewhat agree

c) Agree

d) Strongly agree

1. Using ICT makes me feel uncomfortable

a) Strongly disagree

b) Somewhat agree

c) Agree

d) Strongly agree

1. ICT expands my possibilities and goals

a) Strongly disagree

b) Somewhat agree

c) Agree

d) Strongly agree

1. I don’t need someone to tell me the best way to use ICT

a) Strongly disagree

b) Somewhat agree

c) Agree

d) Strongly agree

1. ICT makes a significant contribution to people’s lives

a) Strongly disagree

b) Somewhat agree

c) Agree

d) Strongly agree

1. I am able to use ICT without the help of other people

a) Strongly disagree

b) Somewhat agree

c) Agree

d) Strongly agree

1. When I use ICT, I am not very sure about what I am doing

a) Strongly disagree

b) Somewhat agree

c) Agree

d) Strongly agree

1. ICT enables me to acquire the important information I need

a) Strongly disagree

b) Somewhat agree

c) Agree

d) Strongly agree

1. ICT makes society more advanced

a) Strongly disagree

b) Somewhat agree

c) Agree

d) Strongly agree

Please indicate how often you usually experience each sensation while working.

1. Prolonged and/or simultaneous use of multiple technological devices to work reduces my concentration and makes me more easily distracted
2. Never
3. Sometimes
4. Often
5. Always

1. Prolonged and/or simultaneous use of multiple technological devices to work negatively affects my performance at work

a) Never

b) Sometimes

c) Often

d) Always

1. Continuous and/or simultaneous use of multiple technological devices to work negatively affects my quality of work life

a) Never

b) Sometimes

c) Often

d) Always

1. Prolonged and/or simultaneous use of multiple technological devices to work causes me additional stress at work

a) Never

b) Sometimes

c) Often

d) Always

1. Due to the enhanced complexity of technological tools, I feel that my workload is increased

a) Never

b) Sometimes

c) Often

d) Always

1. Due to new technologies at work, I feel to be always available for colleagues

a) Never

b) Sometimes

c) Often

d) Always

1. To keep up with new technologies and updates, I have to sacrifice much longer time to work

a) Never

b) Sometimes

c) Often

d) Always

1. Lack of adequate technical and methodological knowledge of devices, software, etc. at work wigging me out

a) Never

b) Sometimes

c) Often

d) Always

1. Technical problems at work result in wasted time and constant interruptions that stressed me a lot

a) Never

b) Sometimes

c) Often

d) Always

1. Frequent use of technological devices to work causes me physical discomfort (migraine, burning eyes, decreased vision, etc.)

a) Never

b) Sometimes

c) Often

d) Always

1. When I work with technological devices, I feel more irritable

a) Never

b) Sometimes

c) Often

d) Always

1. The frequent use of technological devices makes me feel less confident, doubtful, and/or makes it harder for me to make decisions

a) Never

b) Sometimes

c) Often

d) Always

1. Frequent and prolonged use of technological devices to work causes me insomnia and disturbed sleep

a) Never

b) Sometimes

c) Often

d) Always

1. I feel that the use of technological devices at work invades my life too much

a) Never

b) Sometimes

c) Often

d) Always

1. Interacting frequently and for a long time with technological devices causes me feelings of anxiety and tension

a) Never

b) Sometimes

c) Often

d) Always
