# Supplementary material for: Digital wellbeing experience in the workplace: development and validation of the Work-Related Human Computer Interaction Questionnaire
Source: Front Public Health. 2026 Apr 1;14:1778040. doi: 10.3389/fpubh.2026.1778040 (PMC13081781; doi:10.3389/fpubh.2026.1778040)
Supplement: Supplementary file 2 [file Supplementary_file_2.pdf]

Appendix A

Il questionario è composto da affermazioni che descrivono diverse opinioni, comportamenti, atteggiamenti e sensazioni verso gli strumenti informatici e tecnologici (ICT: sia device, ad esempio personal computer, tablet, smartphone; sia tool, ad esempio dashboard di lavoro, strumenti di messaggistica e videochiamata, software aziendali, gestionali, eccetera) sul posto di lavoro

In questa prima sezione ti porremo delle domande per conoscere meglio le tue opinioni su strumenti tecnologici e informatici e il tuo modo di rapportarti con essi durante l'attività lavorativa.

1. Mi sento sicuro/a nel comprendere termini e parole riguardanti il software delle ICT
   a) Per niente
   b) Un po’
   c) Abbastanza
   d) Molto

2. Mi sento sicuro/a nel descrivere le funzionalità dell'hardware delle ICT
   a) Per niente
   b) Un po’
   c) Abbastanza
   d) Molto

3. Mi sento sicuro/a nel risolvere eventuali problemi quando uso le ICT
   a) Per niente
   b) Un po’
   c) Abbastanza
   d) Molto

4. Mi sento sicuro/sicura nella possibilità di comprendere il motivo di un malfunzionamento di un programma sul personal computer
   a) Per niente
   b) Un po’
   c) Abbastanza
   d) Molto

5. Mi sento sicuro/a nell'usare le ICT per raccogliere e/o elaborare dati
   a) Per niente
   b) Un po’
   c) Abbastanza
   d) Molto

6. Mi sento sicuro/a nell'apprendere nuove abilità, così come nuove funzioni e programmi delle ICT
   a) Per niente

b) Un po’
c) Abbastanza
d) Molto

In questa sezione troverai diverse opinioni e modi di sentire verso gli strumenti informatici e tecnologici sul posto di lavoro. Ti chiediamo di dichiarare quanto sei d’accordo con ciascuna di esse.

7. Nella maggior parte dei casi posso imparare da solo/a le cose di cui ho bisogno per usare le ICT
   a) Totalmente in disaccordo
   b) Un po’ d’accordo
   c) Abbastanza d’accordo
   d) Totalmente d’accordo

8. Tendo ad evitare di usare le ICT se posso apparire goffo/a e inesperto/a
   a) Totalmente in disaccordo
   b) Un po’ d’accordo
   c) Abbastanza d’accordo
   d) Totalmente d’accordo

9. Le ICT mi permettono di svolgere il lavoro in modo più interessante e creativo
   a) Totalmente in disaccordo
   b) Un po’ d’accordo
   c) Abbastanza d’accordo
   d) Totalmente d’accordo

10. Ho bisogno di una persona con esperienza che mi aiuti nell’uso delle ICT
   a) Totalmente in disaccordo
   b) Un po’ d’accordo
   c) Abbastanza d’accordo
   d) Totalmente d’accordo

11. Quando devo usare delle ICT ho paura di fare qualche danno irreversibile
   a) Totalmente in disaccordo
   b) Un po’ d’accordo
   c) Abbastanza d’accordo
   d) Totalmente d’accordo

12. Se ho dei problemi mentre uso delle ICT, di solito sono in grado di risolverli da solo/a in un modo o nell’altro
   a) Totalmente in disaccordo
   b) Un po’ d’accordo
   c) Abbastanza d’accordo
   d) Totalmente d’accordo

13. Usare le ICT mi fa sentire a disagio
    a) Totalmente in disaccordo
    b) Un po' d'accordo
    c) Abbastanza d'accordo
    d) Totalmente d'accordo

14. Le ICT ampliano le mie possibilità e i miei obiettivi
    a) Totalmente in disaccordo
    b) Un po' d'accordo
    c) Abbastanza d'accordo
    d) Totalmente d'accordo

15. Non ho bisogno di qualcuno che mi dica qual è il modo migliore per usare le ICT
    a) Totalmente in disaccordo
    b) Un po' d'accordo
    c) Abbastanza d'accordo
    d) Totalmente d'accordo

16. Le ICT danno un grande contributo alla vita delle persone
    a) Totalmente in disaccordo
    b) Un po' d'accordo
    c) Abbastanza d'accordo
    d) Totalmente d'accordo

17. Sono in grado di usare le ICT senza l'aiuto di altre persone
    a) Totalmente in disaccordo
    b) Un po' d'accordo
    c) Abbastanza d'accordo
    d) Totalmente d'accordo

18. Quando uso le ICT non sono molto sicuro/a di ciò che sto facendo
    a) Totalmente in disaccordo
    b) Un po' d'accordo
    c) Abbastanza d'accordo
    d) Totalmente d'accordo

19. Le ICT mi permettono di acquisire le informazioni importanti di cui ho bisogno
    a) Totalmente in disaccordo
    b) Un po' d'accordo
    c) Abbastanza d'accordo
    d) Totalmente d'accordo

20. Le ICT rendono la società più progredita
    a) Totalmente in disaccordo

b) Un po’ d’accordo
c) Abbastanza d’accordo
d) Totalmente d’accordo

In quest’ultima sezione troverai domande che hanno lo scopo di valutare l'impatto dell'utilizzo di strumenti tecnologici e informatici sul tuo benessere psicofisico in ambito lavorativo. Indica per favore con quale frequenza ti capita di provare ciascuna delle sensazioni descritte di seguito, mentre lavori.

21. L'utilizzo prolungato e/o simultaneo di device e tool tecnologici per lavorare riduce il mio livello di concentrazione e mi fa distrarre più facilmente
    a) Mai
    b) Qualche volta
    c) Spesso
    d) Sempre

22. L'utilizzo prolungato e/o simultaneo di device e tool tecnologici per lavorare influisce negativamente sul mio rendimento al lavoro
    a) Mai
    b) Qualche volta
    c) Spesso
    d) Sempre

23. L'uso continuativo e/o simultaneo di device e tool tecnologici per lavorare influisce negativamente sulla mia qualità di vita lavorativa
    a) Mai
    b) Qualche volta
    c) Spesso
    d) Sempre

24. L'utilizzo prolungato e/o simultaneo di device e tool tecnologici per lavorare è per me causa di stress sul lavoro
    a) Mai
    b) Qualche volta
    c) Spesso
    d) Sempre

25. A causa dell’aumentata complessità dei device e dei tool tecnologici, ho l’impressione di aver subito un incremento di carico lavorativo
    a) Mai
    b) Qualche volta
    c) Spesso
    d) Sempre

26. A causa delle nuove tecnologie per lavorare, ho la sensazione di dover essere reperibile per i colleghi e le colleghe
    a) Mai
    b) Qualche volta
    c) Spesso
    d) Sempre

27. Per tenermi al passo con le nuove tecnologie e i continui aggiornamenti, ho la sensazione di dover sacrificare molto più tempo per lavorare
    a) Mai
    b) Qualche volta
    c) Spesso
    d) Sempre

28. La mancanza di conoscenze tecniche e metodologiche adeguate per l'utilizzo di device e tool tecnologici sul lavoro mi manda in crisi
    a) Mai
    b) Qualche volta
    c) Spesso
    d) Sempre

29. I problemi tecnici che possono succedersi mentre lavoro comportano perdite di tempo e continue interruzioni che mi stressano molto
    a) Mai
    b) Qualche volta
    c) Spesso
    d) Sempre

30. L'utilizzo frequente di device e tool tecnologici per lavorare mi causa dei disturbi fisici (emicrania, bruciore agli occhi, calo della vista eccetera)
    a) Mai
    b) Qualche volta
    c) Spesso
    d) Sempre

31. Quando lavoro con device e tool tecnologici mi sento più irritabile
    a) Mai
    b) Qualche volta
    c) Spesso
    d) Sempre

32. Dover utilizzare frequentemente device e tool tecnologici mi fa sentire meno sicuro/a di me, mi fa venire più dubbi e/o mi rende più difficile prendere decisioni
    a) Mai

b) Qualche volta
c) Spesso
d) Sempre

33. L'uso frequente e prolungato di device e tool tecnologici per lavorare mi causa insonnia e sonno disturbato
a) Mai
b) Qualche volta
c) Spesso
d) Sempre

34. Ho la sensazione che l'uso di device e tool tecnologici sul lavoro invada troppo la mia vita
a) Mai
b) Qualche volta
c) Spesso
d) Sempre

35. Interagire frequentemente e a lungo con device e tool tecnologici mi causa sensazioni di ansia e tensione
a) Mai
b) Qualche volta
c) Spesso
d) Sempre
